# Supplementary material for: Cerebrospinal fluid and plasma biomarker trajectories with increasing amyloid deposition in Alzheimer's disease
Source: EMBO Mol Med. 2019 Nov 11;11(12):e11170. doi: 10.15252/emmm.201911170 (PMC6895602; doi:10.15252/emmm.201911170)
Supplement: Supplementary file 1 — Appendix [file EMMM-11-e11170-s001.pdf]

Appendix

Cerebrospinal fluid and plasma biomarker trajectories with increasing amyloid deposition in Alzheimer's disease

Sebastian Palmqvist, MD, PhD, Philip S. Insel, MSc, Erik Stomrud, MD, PhD, Shorena Janelidze, PhD, Henrik Zetterberg, MD, PhD, Britta Brix PhD, Udo Eichenlaub PhD, Jeffrey L. Dage, Xiyun Chai, MD, Kaj Blennow, MD, PhD Niklas Mattsson, MD, PhD, Oskar Hansson MD, PhD.

Table of contents

Appendix Table S1
Appendix Table S2
Appendix Fig. S1
Appendix Fig. S2

Appendix Tables

Appendix Table S1. Inflection points (significant change of 2 SE) for plasma and CSF biomarkers

Biomarker	Significant change (95% CI), SUVR
CSF Aβ40	0.496 (0.452 - 0.539)
Plasma Aβ42	0.537 (0.481 - 0.592)
CSF Aβ42	0.597 (0.582 - 0.611)
Plasma P-tau	0.640 (0.575 - 0.706)
CSF Aβ42/40	0.647 (0.641 - 0.654)
Plasma Aβ42/40	0.680 (0.644 - 0.716)
CSF NfL (early inflection point)	0.697 (0.677 - 0.718)
Plasma NfL	0.706 (0.563 - 0.849)
CSF P-tau	0.718 (0.678 - 0.757)
CSF T-tau	0.739 (0.701 - 0.778)
Plasma T-tau	0.762 (0.622 - 0.902)
CSF Neurogranin	0.804 (0.767 - 0.841)
Hippocampus / intracranial volume	0.813 (0.744 - 0.882)
CSF YKL-40	0.813 (0.711 - 0.915)
CSF NfL (late inflection point)	1.13 (1.11 - 1.15)

Ordered by point on the x-axis (SUVR, see Fig. 1-2). Significant biomarker change was defined as a change of 2 SE from the starting point (left) of the curve. CSF NfL had two distinct inflections (see Fig. 1) and, therefore, two inflection points were calculated. The models for plasma Aβ40 and plasma neurogranin were not significant and thus not included in this inflection analysis. CSF Aβ42, Aβ40, T-tau and P-tau were here analyzed with the Elecsys assay.
Acronyms: NfL, neurofilament light chain; SE, standard error (in this case the standard deviation of the means from the 500 bootstrap samples); SUVR, standardized uptake value ratio.

Appendix Table S2. Performance of the CSF p-tau217 and p-tau181 assays.

Sample	Mean concentration, pg/ml	SD	N	% CV
**CSF P-tau217**				
10 pg/mL QC	9.97	0.59	22	5.95
50 pg/mL QC	44.31	2.06	22	4.66
High Control Sample	737.99	40.96	22	5.55
Medium Control Sample	328.72	17.81	22	5.42
Low Control Sample	59.69	4.93	22	8.26
**CSF P-tau181**				
10 pg/mL QC	9.49	0.24	22	2.53
50 pg/mL QC	45.84	1.05	22	2.29
High Control Sample	379.61	21.25	22	5.60
Medium Control Sample	190.27	7.29	22	3.83
Low Control Sample	71.19	3.92	22	5.51

CV, coefficient of variation; QC, quality control; SD, standard deviation.

Appendix Figures

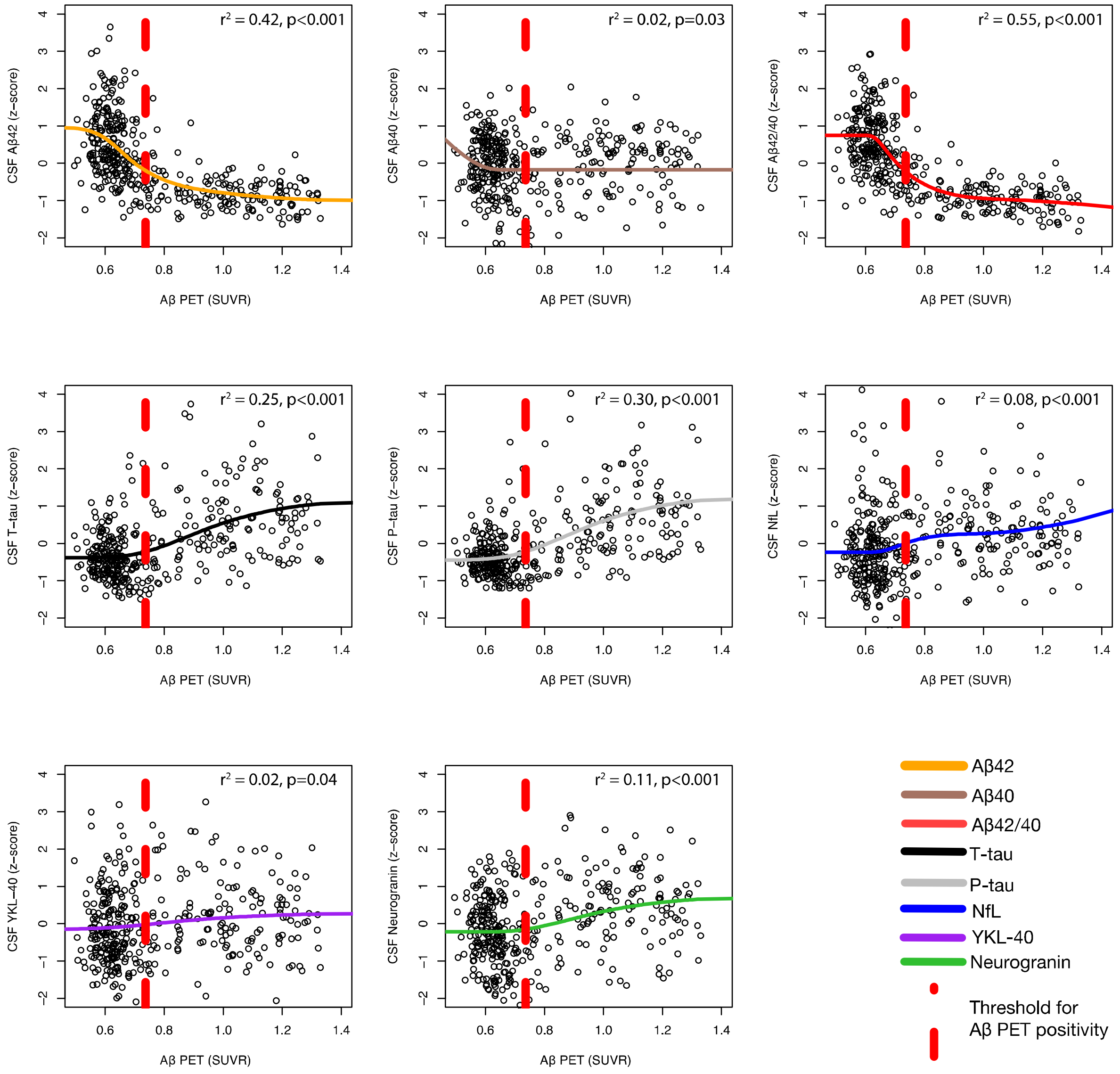

Appendix Figure S1. Monotone spline models of the CSF biomarker trajectories.

P- and r^2 values of the spline models are shown in the upper right corner. One Aβ PET outlier with a SUVR of 1.6959 is not shown in the Figure. Further, the following participants outside the range of the y axis are not shown (x, y coordinates): Aβ40 (1.32, 11.8), NfL (0.79, 4.44), T-tau (1.19, 5.86 and 1.23, 5.76), and P-tau (1.23, 5.81; 1.19, 5.77; and 0.87, 5.04).

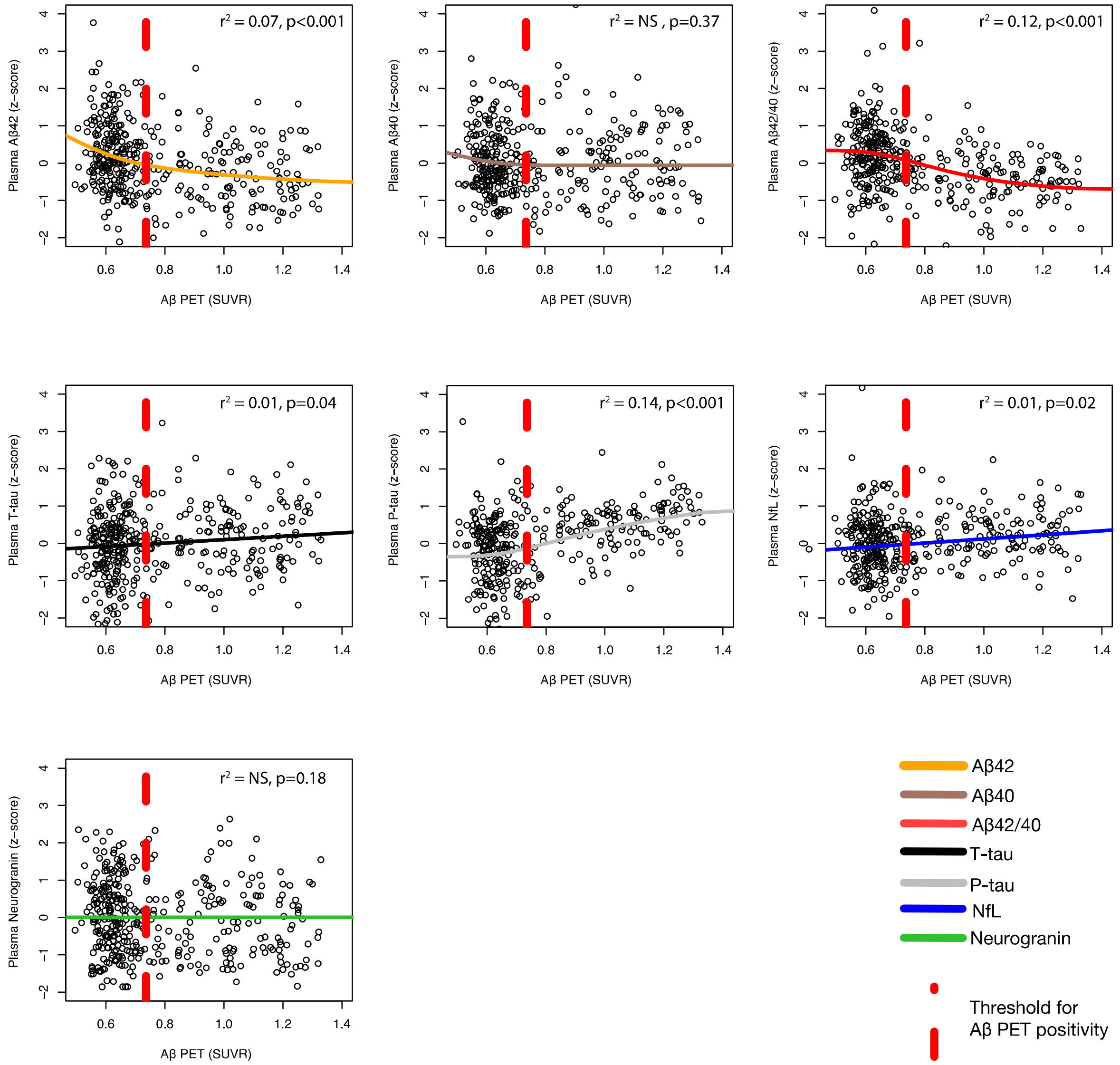

Appendix Figure S2. Monotone spline models of the plasma biomarker trajectories.

P- and r^2 values of the models are shown in the upper right corner. One Aβ PET outlier with a SUVR of 1.6959 is not shown in the Figure. Further, the following participants outside the range of the y axis are not shown (x, y coordinates): Aβ42 (7 participants between -2.39 to -4.26, 0.61 to 1.19), Aβ40 (5 participants between 0.61 to 0.85, -3.02 to -2.37, and 1 at 0.55, 5.48), Aβ42/Aβ40 (6 participants between 0.64 to 1.18, -2.17 to -4.53, 1 at 0.85, 4.69; and 1 at 0.61, 6.08), T-tau (5 participants between 0.62 to 0.77, -2.53 to -2.85 and 1 at 0.55, 4.47); P-tau (7 participants between 0.55 to 1.07, -3.98 to -2.31), NfL (1 at 0.57, -10.8; and 3 between 0.64 to 0.99, -4.06 to 4.96).
